# Machine learning model for predicting improvement in left ventricular systolic function in patients with heart failure and reduced ejection fraction

**DOI:** 10.1016/j.ijcha.2026.101904

**Published:** 2026-03-19

**Authors:** Nariman Sepehrvand, Caitlyn Gilbert, Alec Chunta, Erik Youngson, Justin A. Ezekowitz, Nowell M. Fine, Jonathan G. Howlett, Finlay A. McAlister, Robert J.H. Miller

**Affiliations:** a Division of Cardiology, Department of Cardiac Sciences, Libin Cardiovascular Institute, Cumming School of Medicine, University of Calgary, Calgary, AB, Canada; b Canadian VIGOUR Centre and Department of Medicine, University of Alberta, Edmonton, AB, Canada; c Data and Research Services, Alberta SPOR Support Unit and Provincial Research Data Services, Alberta Health Services, Alberta, Canada

**Keywords:** Machine learning, Gradient boosting, Improved ejection fraction, Heart failure, Left ventricular ejection fraction

## Abstract

**Background:**

About one-third of patients with heart failure (HF) with reduced ejection fraction (HFrEF) may demonstrate left ventricular ejection fraction (LVEF) recovery with medical management. In this study, we developed a machine-learning (ML) model to predict LVEF improvement and compared its performance to a logistic regression (LR) model in internal and external validation cohorts.

**Methods:**

We identified 3124 patients with HFrEF and ≥2 echocardiograms taken ≥6 months apart. Patients were split into development (n = 1812) and external testing (n = 1312) cohorts by site. The ML and LR models were trained using 49 features, with internal 5-fold cross-validation. Prediction performance and calibration in the internal and external testing cohorts were assessed using the area under the curve (AUC) and Brier score, respectively.

**Results:**

LVEF recovery defined as an absolute LVEF increase of ≥10% occurred in 36.0% of the development cohort and 39.8% of the external testing cohort. The ML model performed better in predicting LVEF recovery compared to the LR model in the development cohort (AUC 0.719 vs 0.700, p = 0.045), however, there was no significant difference in external testing (AUC 0.702 vs 0.696, p = 0.498). Lower baseline LVEF and left ventricular dimensions, younger age and non-ischemic etiology contributed the most to the prediction of LVEF improvement.

**Conclusion:**

A ML model using readily available clinical and echocardiographic data did not perform better than traditional LR in predicting LVEF improvement when tested externally. Larger studies, including additional variables or alternative approaches, may improve LVEF recovery prediction.

## 1 Introduction

The recent advances in guideline-directed medical therapy (GDMT) in patients with heart failure (HF), especially in those with HF and reduced ejection fraction (HFrEF) have led to a higher proportion of patients with improved left ventricular (LV) ejection fraction (LVEF) [1]. In certain cases such as in those with myocardial injury due to abnormal metabolic profile and energetics, and toxic or inflammatory insults, this recovery could be spontaneous [2], [3]. There is a direct association between improved LVEF, LV reverse remodeling and improved cardiovascular outcomes in patients with HF [4], [5]. Proportion of patients exhibiting improved LVEF varies considerably between 10% and 40% across published studies due to differing definitions and patient groups (observational real-world cohorts versus clinical trial participants) [6], [7], [8], [9], [10].

Several studies have identified factors associated with LVEF improvement, including younger age, female sex, non-ischemic etiology of heart failure, shorter duration of heart failure, absence of left bundle branch block, having fewer comorbidities, and the use of guideline-directed medical therapies (GDMT) [4], [6], [8], [9], [11], [12], [13], [14], [15]. We previously reported the factors associated with improved LVEF (≥10% absolute improvement in LVEF) over time included younger age, female sex, the presence of hypertension, atrial fibrillation, or cancer, lower baseline LVEF level, and higher hydralazine use [16].

While these studies primarily used traditional statistical methods, there is growing interest in applying machine learning (ML) techniques to predict LVEF improvement. In the current study, we developed and tested a machine-learning model for predicting LVEF improvement in patients with HFrEF and compared its performance to a traditional logistic regression (LR) model.

## 2 Methods

### 2.1 Study population

We included patients with a diagnosis of HF [ICD 10 code I50.x]) between 1 April 2008 and 31 March 2016 with 2 echocardiograms separated by at least 6 months. Patients were included from two separate sites which were split by site into the development (University of Calgary; N = 1812) and external testing (University of Alberta; N = 1312) cohorts (Supplementary Fig. 1) [16]. Baseline covariates were determined using ICD-10-CA codes for all hospitalizations in the year leading up to the index date (date of the initial echocardiogram) [17]. Patients with HFrEF (baseline LVEF < 40) were categorized into two groups based on whether they had improved LVEF on follow-up echocardiogram, defined as absolute LVEF improvement ≥10%. Only two echocardiogram results (baseline and follow-up) were considered in this analysis. The study was approved by the University of Calgary and University of Alberta Research Ethics Boards including waiver of informed consent for use of retrospective data. Our study has been reported according to the Transparent reporting of a multivariable prediction model for individual prognosis or diagnosis based on artificial intelligence (TRIPOD-AI) guidelines [18].

### 2.2 Clinical and echocardiographic data

Demographic data including age, sex, rural versus urban residence, and Pampalon Material Deprivation Index quintiles, which are a neighborhood census-based proxy for socioeconomic status were extracted from administrative databases. Medical history including atrial fibrillation (AF), ischemic heart disease (IHD), diabetes mellitus, hypertension, prior stroke or transient ischemic attack (TIA), chronic kidney disease, chronic obstructive pulmonary disease (COPD), anemia, cancer, dementia, and depression were extracted from the discharge abstract database using validated ICD codes. Medication utilization between echocardiograms including ACEi, ARBs, digoxin, beta-blockers, loop diuretics, MRAs, hydralazine and nitrates were extracted from the Pharmacy Information Network (PIN). PIN captures >95% of community medication dispensations within the province of Alberta. Echocardiography data including LVEF, left ventricular internal diameter end-diastole and end-systole (LVIDD and LVIDS), and mitral regurgitation severity were extracted from echocardiography reports. A summary of variables, including the proportion of missing values, is shown in Supplementary Table 1.

### 2.3 Logistic regression model

A multivariable logistic regression (LR) model was developed using the variables outlined above to predict LVEF improvement. Models were developed using internal 5-fold cross-validation and external testing. For internal cross-validation, the development cohort was randomly split into 5-folds, with four folds (80%) used for model development and one fold (20%) used for internal testing. This process was iterated 5 times until all folds were held out for testing. For external testing, the model was derived in the development cohort and applied to the external population. Associations with LVEF improvement were summarized as adjusted odds ratios (OR) with 95% confidence intervals (CI).

### 2.4 Machine learning model

For the machine learning model, we utilized an ensemble gradient boosted framework − Extreme Gradient Boosting (XGBoost) [19]. Models were developed using internal 5-fold cross-validation and external testing. For internal cross-validation, the development cohort was randomly split into 5-folds, with three folds (60%) used for model training, one fold (20%) for tuning and one fold (20%) used for internal testing. The hyperparameters such as number of training rounds, maximum tree depth, and minimum child weight were tuned based on 5-fold grid-search during the internal cross-validation procedure. For external testing, the model was trained and tuned in the development cohort and applied to the external population.

### 2.5 Model explainability

We utilized two methods for explaining model predictions. First, we utilized feature gain plots which are generated automatically using XGBoost and reflect the importance of features to overall model predictions. Second, we used SHAP (SHapley Additive exPlanations) to graph the overall importance of a variable and its directionality to the final prediction [20].

### 2.6 Statistical analyses

Data are presented as frequencies and percentages for categorical variables, mean ± SD for normally distributed continuous variables, or median (interquartile range) for continuous variables with a skewed distribution. Categorical variables were compared using χ2 or Fisher exact test where appropriate, while continuous variables were compared with *t*-test or Wilcoxon rank sum tests as appropriate. Prediction performance was evaluated using area under the receiver operating characteristic curve (AUC). Calibration was assessed using calibration plots and Brier Score [21].

## 3 Results

### 3.1 Patient populations

Patients in the development cohort were generally older, with a higher proportion of comorbidities including hypertension, diabetes, atrial fibrillation, ischemic heart disease, chronic kidney disease, and chronic obstructive pulmonary disease (COPD) compared to those of the external testing cohort (Table 1). LVEF was slightly lower and LVIDd was larger in the external validation cohort, which had a marginally higher rate of improved LVEF (39.8% vs. 36.0% in the development cohort). Patient with improved LVEF (HFimpEF) were younger, more likely to be female, less likely to have IHD and had lower baseline LVEF and smaller LV dimensions than those with persistent HFrEF (Supplementary Table 2). In the development cohort, the median time between echocardiograms was 1.3 years (interquartile range 0.8 to 2.2 years) and in the external testing cohort it was 1.1 years (interquartile range 0.7 to 2.0 years).

**Table 1:** Baseline characteristics in the development and external testing cohorts.

	Development cohort (N = 1812)	External Testing cohort (N = 1312)	P-value
Age, Median (IQR)	72 (62, 80)	68 (58, 77)	<0.001
Male sex, n(%)	1284 (70.9%)	978 (74.5%)	0.023
Comorbidities, n(%)			
Hypertension	1221 (67.4%)	722 (55.0%)	<0.001
Diabetes	712 (39.3%)	373 (28.4%)	<0.001
IHD	1082 (59.7%)	693 (52.8%)	<0.001
AF	786 (43.4%)	482 (36.7%)	<0.001
Stroke/TIA	150 (8.3%)	86 (6.6%)	0.072
CKD	697 (38.5%)	457 (34.8%)	0.038
COPD	424 (23.4%)	261 (19.9%)	0.019
Cancer	138 (7.6%)	74 (5.6%)	0.030
Anemia	128 (7.1%)	82 (6.3%)	0.37
Dementia	76 (4.2%)	27 (2.1%)	<0.001
Depression	123 (6.8%)	82 (6.3%)	0.55
Medications, n(%)			
ACEi/ARB	1630 (90.0%)	1181 (90.0%)	0.96
Beta-blockers	1650 (91.1%)	1212 (92.4%)	0.19
MRA	747 (41.2%)	643 (49.0%)	<0.001
Digoxin	388 (21.4%)	221 (16.8%)	0.001
Diuretics	1249 (68.9%)	903 (68.8%)	0.95
Nitrates	1066 (58.8%)	153 (11.7%)	<0.001
Hydralazine	170 (9.4%)	31 (2.4%)	<0.001
Echocardiogram			
Baseline LVEF, median (IQR)	30 (25, 35)	27.5 (19, 32)	<0.001
LVIDd, median (IQR)	5.66 (5.05, 6.20)	5.83 (5.29, 6.40)	<0.001
LVIDs, median (IQR)	4.84 (4.12, 5.58)	4.55 (3.85, 5.38)	<0.001
Improved LVEF, n(%)	652 (36.0%)	522 (39.8%)	0.030

ACEi: angiotensin-converting enzyme inhibitors; AF: atrial fibrillation; ARB: angiotensin receptor blocker; CKD: chronic kidney disease; COPD: chronic obstructive pulmonary disease; IHD: ischemic heart disease; IQR: interquartile range; LVEF: left ventricular ejection fraction; LVIDd: left ventricular internal diameter at end diastole; LVIDs: left ventricular internal diameter at end systole; MRA: mineralocorticoid receptor antagonists; N: number; TIA: transient ischemic attack.

### 3.2 Logistic regression model

Variables significantly and independently associated with improved LVEF included: younger age (aOR 0.98, 95%CI 0.97–0.99 per year), inpatient versus outpatient diagnosis (aOR 0.48, 95%CI 0.34–0.67), comorbidities such as hypertension (aOR 1.41, 95%CI 1.09–1.81), AF (aOR 1.28, 95%CI 1.02–1.60), CKD (aOR 1.37, 95%CI 1.09–1.72), and cancer (aOR 1.58, 95%CI 1.07–2.34), MRA use (aOR 1.30, 95%CI 1.02–1.65), hydralazine use (aOR 1.79, 95%CI 1.19–2.67), baseline LVEF (aOR 0.89, 95%CI 0.87–0.90 per 5%), baseline LVIDd (aOR 0.77, 95%CI 0.62–0.96 per centimeter), and baseline LVIDs (aOR 0.73, 95%CI 0.60–0.89 per centimeter). (Supplementary Table 3).

### 3.3 XGB model: Internal validation

The machine learning model was trained on 1812 patients with HFrEF with follow-up echocardiograms. LVEF improvement was observed in 652 (36%) of the patients in the development cohort. The machine learning model had higher prediction performance (AUC 0.719, 95%CI 0.695 – 0.744) compared to the logistic regression model (AUC 0.700, 95% CI 0.675 – 0.725) (p = 0.045) in the development cohort.

### 3.4 External validation

**Fig. 1:**
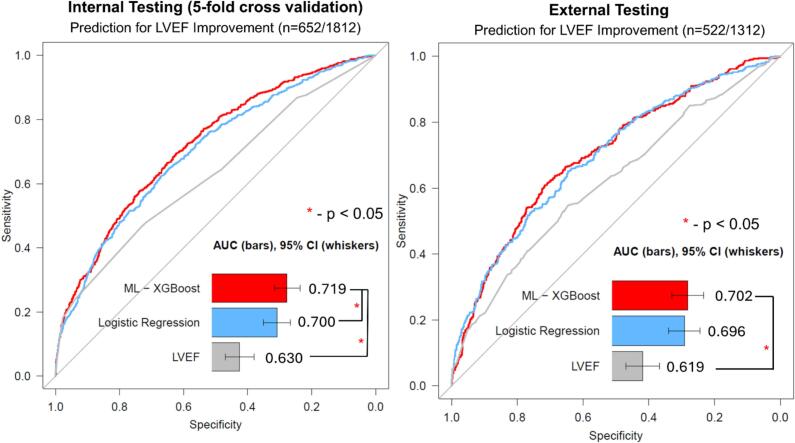
The predictive performance of XGBoost model in predicting improved LVEF in patients with HFrEF in the development/internal testing (Panel A) and external testing (Panel B) datasets, when compared to a logistic regression model developed based on baseline characteristics. AUC: area under the receiver operator curve, CI: confidence interval, HFrEF: heart failure with reduced ejection fraction; LVEF: left ventricular ejection fraction, ML: machine learning.

**Fig. 2:**
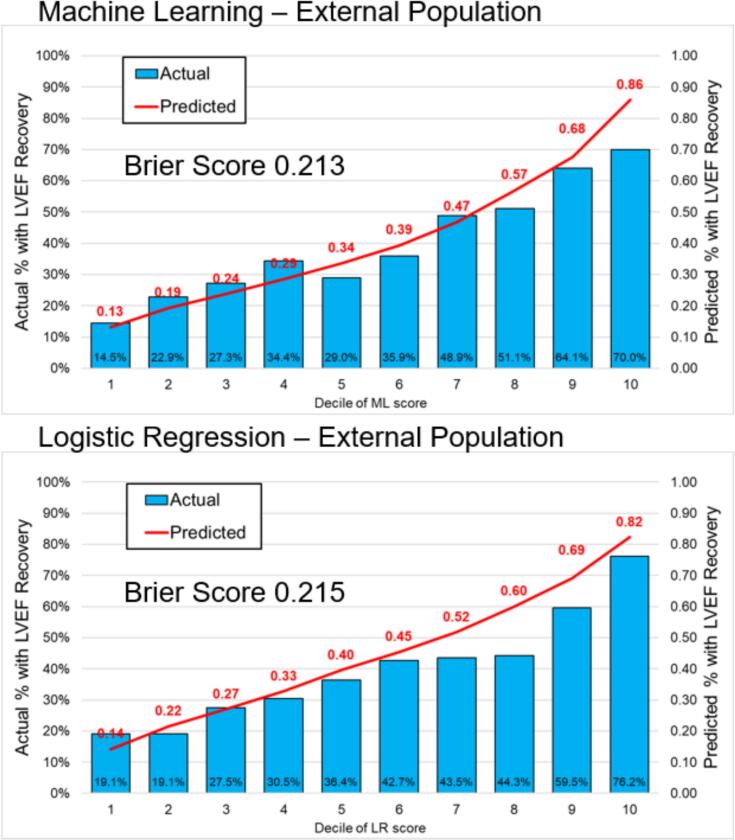
Calibration in External Testing Population. LVEF: left ventricular ejection fraction, LR: logistic regression, ML: machine learning.

The model was externally tested on 1312 patients. Improved EF was observed in 522 (39.8%) of these patients in follow-up echocardiograms. The XGBoost model showed an AUC of 0.702 (95%CI 0.673 – 0.731) in the external testing cohort, which was similar compared to logistic regression (AUC 0.696, 95% CI 0.667 – 0.725) (p = 0.592) (Fig. 1). Similarly, there was no difference in calibration (Fig. 2) with Brier scores of 0.213 for machine learning and 0.215 for logistic regression.

### 3.5 Model visualization

**Fig. 3:**
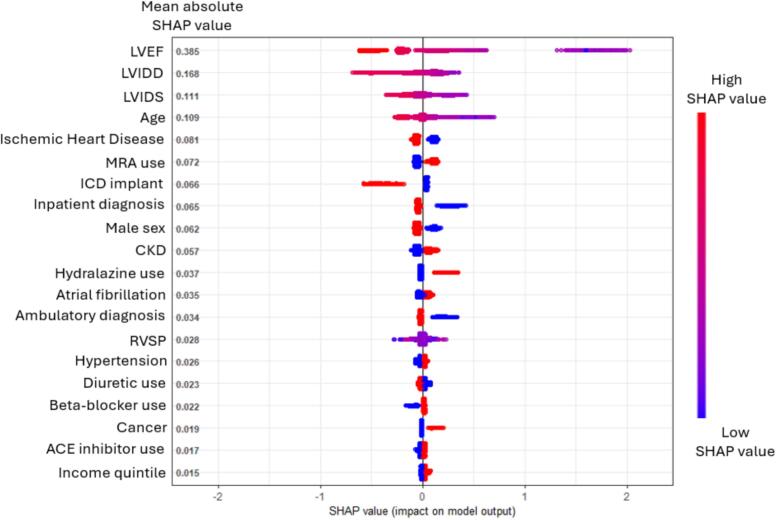
SHapley Additive exPlanations (SHAP) analysis of the XGBoost model. ACE: angiotensin-converting enzyme; CKD: chronic kidney disease; ICD: implantable cardioverter defibrillator; LVEF: left ventricular ejection fraction; LVIDd and LVIDs: Left ventricular internal diameter end-diastole and end-systole; MRA: mineralocorticoid receptor antagonists; RVSP: right ventricular systolic pressure;

The SHAP analysis revealed that the top contributors to LVEF improvement prediction in the XGBoost model were lower baseline LVEF, lower baseline left ventricular dimensions, younger, non-ischemic HF etiology, and female sex. These factors played significant roles in determining the likelihood of ejection fraction improvement in patients with heart failure with reduced ejection fraction (HFrEF) (Fig. 3 and Supplementary Fig. 2).

## 4 Discussion

In this study, we developed and validated an ML model aimed at predicting improvement in LVEF in patients with HFrEF. The ML model showed moderate prediction performance in internal and external testing. However, the ML model did not outperform the LR model in the external testing dataset. Furthermore, calibration was similar for the ML and LR models. To our knowledge, no prior study has externally tested and directly compared ML and logistic regression models for predicting LVEF improvement. Our work highlights the importance of external testing when evaluating ML models.

It is imperative to discern the cohort of patients with HFrEF exhibiting an improved LVEF from individuals with HF with preserved ejection fraction (HFpEF) or those with HF with mid-range LVEF (HFmrEF) as the former alludes to the trajectory of LVEF changes usually in response to GDMT and device therapy and the latter two are alluding to HF subgroups based on LVEF categories. Kalogeropoulos et al. reported age and sex-adjusted mortality to be 16.3%, 13.2%, and 4.8% in patients with HFrEF, HFpEF, and HFimpEF, respectively [22]. Furthermore, patients with HFimpEF displayed a reduced risk of composite events in comparison to those with HFrEF and HF with mid-range but no improved EF in a study of patients with HF undergoing cardiopulmonary exercise testing [23].

In the multivariable regression analysis, younger age, non-ischemic HF etiology, comorbidities such as hypertension, AF, CKD, and cancer, use of MRA or hydralazine, lower baseline LVEF, and smaller LV dimensions were associated with higher odds of LVEF recovery in patients with HFrEF. These mirror the findings of numerous previous studies [8], [9], [14], [24], [25]. We found the baseline LVEF and LV dimensions to be top contributors to the model’s prediction of LVEF recovery. Given the importance of LV reverse remodeling in LVEF recovery, and the association of LVEF improvement with a reciprocal decrease in LV end-diastolic volume, it is expected that baseline LV function and dimensions play an important role in predicting LVEF recovery [26], [27].

A scientific expert panel in 2020 attempted to achieve a consensus to address the heterogeneity in defining LVEF recovery. This definition, proposed by the Journal of American College of Cardiology Scientific Expert Panel [7], included three key criteria: (1) a baseline LVEF < 40%, indicating reduced baseline systolic function; (2) an absolute improvement in LVEF of at least 10%; and (3) a subsequent LVEF >40% on follow-up assessment. Despite this effort, there remains a significant variability across clinical guidelines. The 2021 ESC Guideline defined recovered LVEF as patients with a prior LVEF≤40% who later present with an LVEF ≥50% [28]. However, the 2022 AHA/ACC/HFSA HF guideline defined HFimpEF as a prior LVEF ≤40% with a follow-up LVEF >40% [1]. In our study, we adopted a modified version of the JACC expert panel’s definition [7]. Given the well-documented inter-observer and intra-observer variability in echocardiographic LVEF measurements of approximately ±5%, we considered a >10% absolute improvement in LVEF to be a more robust indicator of true physiological recovery. However, we did not impose a strict requirement for follow-up LVEF to exceed 40%, since improvement from 39% to 41% would not likely be clinically significant.

In our study, the ML model did not outperform traditional LR models in external testing. This observation suggests that larger datasets, incorporating a broader range of candidate variables, would be needed to realize the benefits of machine learning. This is not the first instance where ML models have demonstrated limited or no improvement in predictive performance compared to traditional LR models. Christodoulou et al. reviewed 71 studies comparing ML models versus LR for clinical predictions and concluded that ML showed no performance superiority over LR under low bias conditions [29]. Numerous studies have previously demonstrated that simply using a more complex algorithm does not necessarily lead to improved predictions if predictions are based on the same variables [30], [31], [32]. Similar findings were reported from HF [33], [34] and non-HF [35], [36] settings. A systematic review of studies using machine learning methods compared with conventional statistical models for predicting readmission and mortality in patients with HF showed that the majority of studies using machine learning were not externally validated, and calibration was rarely assessed [37].

This study has several limitations. Our study lacked some relevant clinical covariates such as natriuretic peptides or functional status at baseline. Previous studies have shown that a more pronounced decrement in natriuretic peptide levels with GDMT correlates with enhanced LVEF and a more substantial reduction in left ventricular (LV) volumes, along with ameliorated clinical outcomes [38]. Our study investigated repeat echocardiograms in a ≥6-month time period to assess for interval changes in cardiac function. Longer follow-ups after identifying those with LVEF recovery are needed to assess the durability of recovery and factors associated with this in the future. Although we had access to the medication data in this study, given the study period, there was no treatment with angiotensin receptor/neprilysin inhibitors or sodium-glucose co-transporter inhibitors in our patient population. This may limit the generalizability of our findings to contemporary clinical practice. Despite demographic and clinical differences between the internal and external validation cohorts, the machine learning model performed consistently, suggesting model robustness. However, the AUC values near 0.7 indicate room for improvement, possibly by expanding the input baseline variables, refining feature selection, or using more advanced algorithms. We previously have shown the added value of using deep learning analysis of the actual echo images as compared to utilizing the echocardiographer-driven measurements [39].

In conclusion, the machine learning model using readily available clinical and echocardiographic data showed moderate predictive performance for LVEF improvement in patients with HFrEF but did not perform better than traditional LR in the external validation cohort. This highlights the importance of external validation and following quality standards for prognosis research. Future work may be able to improve the model's discriminative power by exploring additional clinical features such as biomarkers or incorporating direct image analysis.

## 5 Authorship

All authors take responsibility for all aspects of the reliability and freedom from bias of the data presented and their discussed interpretation.

## CRediT authorship contribution statement

**Nariman Sepehrvand:** Writing – original draft, Investigation, Conceptualization. **Caitlyn Gilbert:** Writing – review & editing, Formal analysis, Conceptualization. **Alec Chunta:** Writing – review & editing, Investigation, Formal analysis. **Erik Youngson:** Writing – review & editing, Investigation, Formal analysis, Data curation. **Justin A. Ezekowitz:** Writing – review & editing, Data curation, Conceptualization. **Nowell M. Fine:** . **Jonathan G. Howlett:** Writing – review & editing, Data curation, Conceptualization. **Finlay A. McAlister:** Writing – review & editing, Investigation, Conceptualization. **Robert J.H. Miller:** Writing – review & editing, Supervision, Formal analysis, Data curation, Conceptualization.

## Declaration of competing interest

NF has received research support and consulting honoraria from Pfizer, Alnylam, Eidos/BridgeBio, AstraZeneca, Ionis, NovoNordisk, Janssen, and Sanofi. RM received research support from Alberta Innovates and consulting support from Bayer and Alnylam. All the other authors have no relevant conflicts of interest to disclose.
