# Supplementary material for: Machine learning model for predicting improvement in left ventricular systolic function in patients with heart failure and reduced ejection fraction
Source: Int J Cardiol Heart Vasc. 2026 Mar 19;64:101904. doi: 10.1016/j.ijcha.2026.101904 (PMC13018860; doi:10.1016/j.ijcha.2026.101904)
Supplement: Supplementary Data 1 [file mmc1.docx]

**SUPPLEMENTARY MATERIAL:**

**Supplementary Table 1:** Proportion of Missing Values for the Variables included in the model

| Variable | % missing,  internal cohort | % missing,  external testing cohort |
| --- | --- | --- |
| Age | 0.00% | 0.00% |
| Sex | 0.00% | 0.00% |
| Income quintile | 1.77% | 2.97% |
| Rural | 0.00% | 0.00% |
| Diagnosis location | 0.00% | 0.00% |
| Hypertension | 0.00% | 0.00% |
| Diabetes | 0.00% | 0.00% |
| IHD | 0.00% | 0.00% |
| AF | 0.00% | 0.00% |
| Stroke/TIA | 0.00% | 0.00% |
| CKD | 0.00% | 0.00% |
| COPD | 0.00% | 0.00% |
| Cancer | 0.00% | 0.00% |
| Anemia | 0.00% | 0.00% |
| Dementia | 0.00% | 0.00% |
| Depression | 0.00% | 0.00% |
| ACEi/ARB | 0.00% | 0.00% |
| Beta-blockers | 0.00% | 0.00% |
| MRA | 0.00% | 0.00% |
| Digoxin | 0.00% | 0.00% |
| Diuretics | 0.00% | 0.00% |
| Nitrates | 0.00% | 0.00% |
| Hydralazine | 0.00% | 0.00% |
| Pacemaker between 2 echos | 0.00% | 0.00% |
| ICD between 2 echos | 0.00% | 0.00% |
| CRT-P between 2 echos | 0.00% | 0.00% |
| Baseline LVEF | 0.00% | 0.00% |
| LVIDd | 6.40% | 3.20% |
| LVIDs | 10.70% | 5.26% |
| MR severity | 14.33% | 8.46% |

ACEi: angiotensin-converting enzyme inhibitor; AF: atrial fibrillation; ARB: angiotensin receptor blocker; CKD: chronic kidney disease; CRT-P: Cardiac resynchronization therapy-Pacemaker; COPD: chronic obstructive pulmonary disease; ICD: implantable cardioverter defibrillator; IHD: ischemic heart disease; LVEF: left ventricular ejection fraction; LVIDd and LVIDs: Left ventricular internal diameter end-diastole and end-systole; MR: mitral regurgitation; MRA: mineralocorticoid receptor antagonists;

**Supplementary Table 2.** Baseline characteristics between groups with and without recovered LVEF

|  | HF recovered EF  N=1174 | Not HF recovered EF  N=1950 | P-value |
| --- | --- | --- | --- |
| Age, Median (IQR) | 68 (58, 78) | 71 (61, 79) | <0.001 |
| Male sex, n(%) | 798 (68.0%) | 1464 (75.1%) | <0.001 |
| **Comorbidities**, n(%) |  |  |  |
| Hypertension | 738 (62.9%) | 1205 (61.8%) | 0.55 |
| Diabetes | 398 (33.9%) | 687 (35.2%) | 0.45 |
| IHD | 601 (51.2%) | 1174 (60.2%) | <0.001 |
| AF | 508 (43.3%) | 760 (39.0%) | 0.018 |
| Stroke/TIA | 86 (7.3%) | 150 (7.7%) | 0.71 |
| CKD | 460 (39.2%) | 694 (35.6%) | 0.044 |
| COPD | 257 (21.9%) | 428 (21.9%) | 0.97 |
| Cancer | 91 (7.8%) | 121 (6.2%) | 0.096 |
| Anemia | 78 (6.6%) | 132 (6.8%) | 0.89 |
| Dementia | 27 (2.3%) | 76 (3.9%) | 0.015 |
| Depression | 87 (7.4%) | 118 (6.1%) | 0.14 |
| **Medications**, n(%) |  |  |  |
| ACEi/ARB | 995 (84.8%) | 1645 (84.4%) | 0.77 |
| Beta-blockers | 1044 (88.9%) | 1700 (87.2%) | 0.15 |
| MRA | 486 (41.4%) | 730 (37.4%) | 0.028 |
| Digoxin | 214 (18.2%) | 320 (16.4%) | 0.19 |
| Diuretics | 688 (58.6%) | 1173 (60.2%) | 0.39 |
| Nitrates | 371 (31.6%) | 662 (33.9%) | 0.18 |
| Hydralazine | 72 (6.1%) | 88 (4.5%) | 0.047 |
| **Echo data** |  |  |  |
| Baseline LVEF, median (IQR) | 25 (17.5, 35) | 30 (25, 35) | <0.001 |
| LVIDd, median (IQR) | 5.6 (5.0, 6.2) | 5.8 (5.2, 6.4) | <0.001 |
| LVIDs, median (IQR) | 4.6 (3.9, 5.4) | 4.7 (4.0, 5.5) | 0.005 |

ACEi: angiotensin-converting enzyme inhibitors; AF: atrial fibrillation; ARB: angiotensin receptor blocker; CKD: chronic kidney disease; COPD: chronic obstructive pulmonary disease; IHD: ischemic heart disease; IQR: interquartile range; LVEF: left ventricular ejection fraction; LVIDd: left ventricular internal diameter at end diastole; LVIDs: left ventricular internal diameter at end systole; MRA: mineralocorticoid receptor antagonists; N: number; TIA: transient ischemic attack.

**Supplementary Table 3:** Multivariable logistic regression model

| Variable | OR (95% CI) | p-values |
| --- | --- | --- |
| Age | 0.98 (0.97 - 0.99) | <0.001 |
| Male sex | 0.80 (0.63 - 1.02) | 0.071 |
| Income quintile |  |  |
| 1 (highest) | Reference | Reference |
| 2 | 0.78 (0.55 - 1.10) | 0.156 |
| 3 | 1.14 (0.81 - 1.60) | 0.46 |
| 4 | 0.97 (0.69 - 1.35) | 0.839 |
| 5 (lowest) | 0.93 (0.66 - 1.31) | 0.668 |
| Missing | 1.55 (0.68 - 3.53) | 0.297 |
| Rural residence | 0.71 (0.48 - 1.05) | 0.086 |
| Inpatient Diagnosis | 0.48 (0.34 - 0.67) | <0.001 |
| Hypertension | 1.41 (1.09 - 1.81) | 0.009 |
| Diabetes | 0.94 (0.75 - 1.19) | 0.629 |
| IHD | 0.70 (0.55 - 0.88) | 0.002 |
| AF | 1.28 (1.02 - 1.60) | 0.031 |
| Stroke/TIA | 0.90 (0.61 – 1.34) | 0.609 |
| CKD | 1.37 (1.09 - 1.72) | 0.008 |
| COPD | 0.97 (0.75 - 1.25) | 0.794 |
| Cancer | 1.58 (1.07 - 2.34) | 0.021 |
| Anemia | 1.07 (0.70 - 1.62) | 0.767 |
| Dementia | 0.56 (0.31 - 1.01) | 0.055 |
| Depression | 0.81 (0.53 - 1.24) | 0.328 |
| ACEi/ARB | 1.10 (0.87 - 1.40) | 0.411 |
| Beta-blockers | 1.35 (0.95 - 1.93) | 0.093 |
| MRA | 1.30 (1.02 - 1.65) | 0.037 |
| Digoxin | 1.02 (0.77 - 1.36) | 0.892 |
| Diuretics | 0.83 (0.64 - 1.06) | 0.135 |
| Nitrates | 0.99 (0.78 - 1.26) | 0.959 |
| Hydralazine | 1.79 (1.19 - 2.67) | 0.005 |
| Pacemaker between | 0.89 (0.55 - 1.46) | 0.654 |
| ICD between | 0.39 (0.25 - 0.60) | <0.001 |
| CRT-P between | 0.67 (0.28 - 1.61) | 0.374 |
| Baseline LVEF | 0.89 (0.87 - 0.90) | <0.001 |
| LVIDd | 0.77 (0.62 - 0.96) | 0.022 |
| LVIDs | 0.73 (0.60 - 0.89) | 0.001 |
| MR severity |  |  |
| Trace/none | Reference | Reference |
| Mild | 1.01 (0.75 - 1.36) | 0.966 |
| Mild-moderate | 1.05 (0.72 - 1.53) | 0.789 |
| Moderate | 0.99 (0.68 - 1.44) | 0.944 |
| Moderate-severe | 0.93 (0.58 - 1.52) | 0.783 |
| Severe | 1.06 (0.53 - 2.10) | 0.871 |

ACEi: angiotensin-converting enzyme inhibitors; AF: atrial fibrillation; ARB: angiotensin receptor blocker; CI: confidence interval; CKD: chronic kidney disease; COPD: chronic obstructive pulmonary disease; IHD: ischemic heart disease; IQR: interquartile range; LVEF: left ventricular ejection fraction; LVIDd: left ventricular internal diameter at end diastole; LVIDs: left ventricular internal diameter at end systole; MR: mitral regurgitation; MRA: mineralocorticoid receptor antagonists; N: number; OR: odds ratio; TIA: transient ischemic attack;

**Supplementary Figure 1.** Flowchart of the study cohorts for the model training and external validation


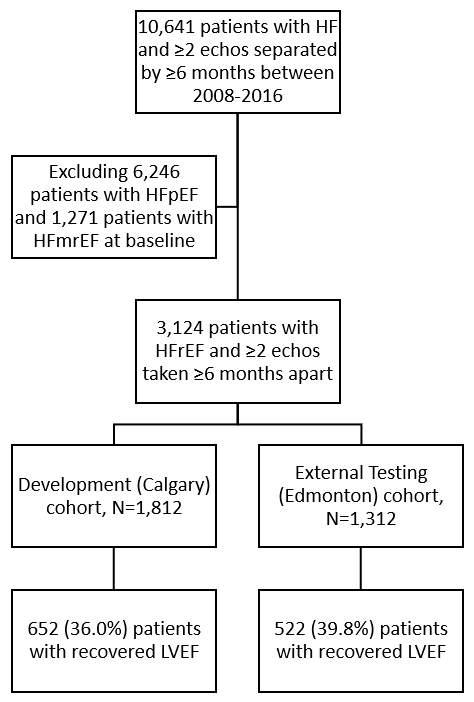


HF: heart failure; HFmrEF: HF with mildly-reduced ejection fraction; HFpEF: HF with preserved ejection fraction; HFrEF: HF with reduced ejection fraction; LVEF: left ventricular ejection fraction; N: number

**Supplementary Figure 2.** Information gain ranking of features in the XGBoost model
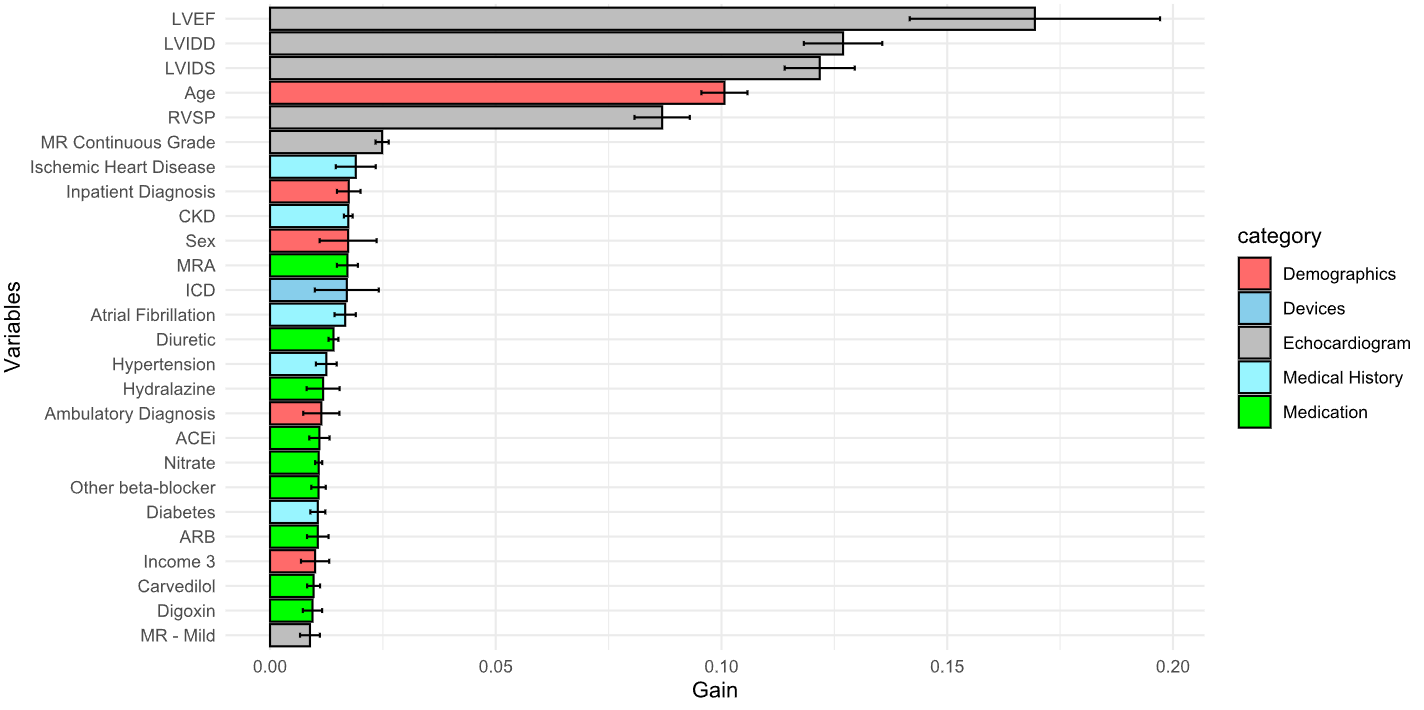


ACE: angiotensin-converting enzyme; CKD: chronic kidney disease; ICD: implantable cardioverter defibrillator; LVEF: left ventricular ejection fraction; LVIDd and LVIDs: Left ventricular internal diameter end-diastole and end-systole; MRA: mineralocorticoid receptor antagonists; RVSP: right ventricular systolic pressure.
